# Sticker-and-spacer model for amyloid beta condensation and fibrillation

**DOI:** 10.3389/fnmol.2022.962526

**Published:** 2022-10-13

**Authors:** Jack P. Connor, Steven D. Quinn, Charley Schaefer

**Affiliations:** ^1^Department of Biology, University of York, York, United Kingdom; ^2^School of Physics, Engineering and Technology, University of York, York, United Kingdom; ^3^Astbury Centre for Structural Molecular Biology, School of Molecular and Cellular Biology, University of Leeds, Leeds, United Kingdom; ^4^York Biomedical Research Institute, University of York, York, United Kingdom

**Keywords:** Alzheimer's disease, amyloid, aggregation, condensates, liquid-liquid phase separation, single-molecule, bond-fluctuation model

## Abstract

A major pathogenic hallmark of Alzheimer's disease is the presence of neurotoxic plaques composed of amyloid beta (Aβ) peptides in patients' brains. The pathway of plaque formation remains elusive, though some clues appear to lie in the dominant presence of Aβ_1 − 42_ in these plaques despite Aβ_1−40_ making up approximately 90% of the Aβ pool. We hypothesize that this asymmetry is driven by the hydrophobicity of the two extra amino acids that are incorporated in Aβ_1−42_. To investigate this hypothesis at the level of single molecules, we have developed a molecular “sticker-and-spacer lattice model” of unfolded Aβ. The model protein has a single sticker that may reversibly dimerise and elongate into semi-flexible linear chains. The growth is hampered by excluded-volume interactions that are encoded by the hydrophilic spacers but are rendered cooperative by the attractive interactions of hydrophobic spacers. For sufficiently strong hydrophobicity, the chains undergo liquid-liquid phase-separation (LLPS) into condensates that facilitate the nucleation of fibers. We find that a small fraction of Aβ_1−40_ in a mixture of Aβ_1−40_ and Aβ_1−42_ shifts the critical concentration for LLPS to lower values. This study provides theoretical support for the hypothesis that LLPS condensates act as a precursor for aggregation and provides an explanation for the Aβ_1−42_-enrichment of aggregates in terms of hydrophobic interactions.

## 1. Introduction

Alzheimer's disease (AD), the most common cause of dementia (60–80% of cases Abeysinghe et al., 2020), and is a fatal neurodegenerative disease causing severe and devastating cognitive impairment. Age is the biggest risk factor for AD, of which, most cases are sporadic (around 95%) and occur over the age of 65 (Abeysinghe et al., 2020; Zhang et al., 2020; Zhao et al., 2020; Ayodele et al., 2021). The exact cause of AD is not fully understood and with better living conditions meaning average life expectancy in developed countries is increasing, the number of cases and the burden of neurodegenerative disease is increasing worldwide. As of 2018, an estimated 50 million people worldwide suffer from dementia, with this number expected to triple by 2050 (Patterson, 2018).

A major pathogenic hallmark in AD brains is the presence of extracellular neurotoxic plaques made up of amyloid beta (Aβ) (Stelzmann et al., 1995; Breijyeh and Karaman, 2020). Aβ is produced from amyloid precursor protein (APP) which is cleaved by α, β and γ secretases (Chen et al., 2017; Guo et al., 2020; Hampel et al., 2021). APP proteolytic cleavage can be separated into non-amyloidogenic and amyloidogenic pathways. We will focus on the amyloidogenic pathway as it is relevant to AD. First APP is cleaved by β secretase into soluble APPβ and a 99 amino acid C-terminal fragment (C99). C99 is then cleaved by γ secretase at multiple sites giving rise to Aβ of multiple lengths ranging from 39-51 amino acids (Zhang et al., 2011; Haass et al., 2012). After cleavage, Aβ is secreted from the cell where it can then form oligomers, fibrils, and finally plaques. The amyloid cascade hypothesis suggests that the deposition of Aβ into senile plaques is critical for AD pathology (Hardy and Higgins, 1992; Karran et al., 2011; Castellani et al., 2019) but the exact mechanism and change in AD brains that lead to this neurotoxic aggregation is not fully understood. Recently, this hypothesis has been called into question based on amounting evidence in disagreement. Amyloid deposition does not correlate with neuronal loss (Kametani and Hasegawa, 2018) and amyloid burden can be identified in cognitively unimpaired individuals (Arenaza-Urquijo and Vemuri, 2018; Dubois et al., 2021). Furthermore, clinical trials using therapeutics that target Aβ for degradation have been ineffective thus far (Ricciarelli and Fedele, 2017). A particular issue for anti-Aβ therapeutics is that most treatments focus on clearing insoluble aggregates (Tolar et al., 2019) with increasing evidence suggesting that pre-fibrillar soluble oligomers are orders of magnitude more toxic than fibrils and plaques (Chafekar et al., 2008; Ono et al., 2009; Sengupta et al., 2016; Huang and Liu, 2020). Unlike plaques, oligomers have been shown to impair both synaptic function and structure (Selkoe and Hardy, 2016; Kametani and Hasegawa, 2018). A recent study by Ghadami et al. (2020) has demonstrated that transthyretin is neuroprotective which is achieved by binding to Aβ oligomers, inhibiting primary and secondary nucleation without altering elongation, ephasising the role of oligomers in Aβ-mediated neurotoxicity. Consequently, understanding the role of pre-fibrillar Aβ species such as soluble oligomers and their contribution to AD pathology have gained significant interest in recent years.

The most predominant Aβ species is 40 amino acids long (Aβ_1−40_) and makes up ~90% of the Aβ pool. A less prevalent 42 amino acid long species of Aβ (Aβ_1−42_), which accounts for ~10 % of the Aβ population is of particular importance to AD due to a higher aggregation propensity. Compared to Aβ_1−40_, Aβ_1−42_ has a longer chain length that increases a repulsive excluded-volume interaction but adds an attractive hydrophobic interaction due to the addition of two C-terminal hydrophobic amino acids. As such, Aβ_1−42_ has increased aggregation propensity and is a predominant component of senile plaques (Iwatsubo et al., 1994; Tamaoka et al., 1994; Gu and Guo, 2021). Familial AD mutations in APP, PSEN1, and PSEN2, cause increased production of Aβ, with abnormally high levels of Aβ_1−42_ relative to Aβ_1−40_ (Scheuner et al., 1996; Hecimovic et al., 2004; Kumar-Singh et al., 2006; Chávez-Gutiérrez and Szaruga, 2020). Recent work shows that the ratio of Aβ_1−42_:Aβ_1−40_ in plasma and cerebrospinal fluid can be used as a biomarker for AD diagnosis (Baldeiras et al., 2018; Lehmann et al., 2018; Nakamura et al., 2018; Zetterberg, 2019; Doecke et al., 2020; Mahaman et al., 2022; Teunissen et al., 2022) as it correlates with Aβ deposition (Fandos et al., 2017) and cognitive decline (Yaffe et al., 2011; Pérez-Grijalba et al., 2019; Giudici et al., 2020; Lim et al., 2020). This evidence suggests that an increased ratio of Aβ_1−42_:Aβ_1−40_ may promote the formation of neurotoxic aggregation and the progression of AD.

Hence, in summary, while the physico-chemical properties of Aβ remains subject to debate, these properties appear to correlate with the structural properties of the aggregates. In this study, we focus our attention on the pathway of self-assembly (Michaels et al., 2020), and aim to understand how this pathway is affected by the molecular properties of Aβ, such as hydrophobicity and intrinsic disorder. We hypothesize that when Aβ_1−42_ interacts with Aβ_1−40_ sufficient hydrophobicity is added to enhance self-assembly, while the excluded volume interaction is limited. We will model this on a molecular level by describing the intrinsically disordered protein as chains with hydrophilic and hydrophobic segments, as well as ‘sticky' building blocks that may reversibly self-ensemble into needle-like aggregates.

The aggregation of intrinsically disordered poly-peptides (IDPs) is an important topic in the biological physics of vital intracellular and extracellular processes. In particular, this class of proteins is known to undergo liquid-liquid phase separation (LLPS) to serve biological functionality, such as the (temporary) formation of membraneless organelles (Shin and Brangwynne, 2017; Jin et al., 2021), but also appears responsible to form large droplet-like precursors for the aggregation of the tau protein (Wegmann et al., 2018), which is another hallmark for AD. Large structures (possibly micellar) of approximately 50 Aβ molecules that are speculated to facilitate Aβ aggregation have also been observed (Wegmann et al., 2018). In the present study, we analyse the self-assembly of single peptides to address the current lack of physical models that explain both the formation of large agglomerates and the transition into fibrillar structures in terms of the molecular properties of the IDPs.

We argue that the general mechanism of coupled LLPS and aggregation is an example of physics that emerges from simple concepts such as (non-specific) hydrophobic interactions, excluded volume, and specific interactions. This is a hopeful scenario, as it implies that we may gain relevant molecular insight into this mechanism using strongly coarse-grained molecular models, rather than full atomistic molecular-dynamics simulations that are computationally too demanding to reach the relevant timescales. A commonly used coarse-graining approach to capture both the polymer physics of the polypeptide and the localized formation of reversible non-covalent bonds is to develop a sticker-and-spacer model. Such models were originally developed for synthetic associating polymers (Leibler et al., 1991), and were later adopted for disordered, multivalent proteins, including natural silk (Schaefer et al., 2020; Schaefer and McLeish, 2022) and scaffolding proteins in biological condensates (Shin and Brangwynne, 2017 and Choi et al., 2019, 2020). Typical simulation approaches solve the Brownian dynamics of the proteins using simulation software packages such as MARTINI or LAMMPS. A major challenge, which is receiving wide attention from the modeling community (Gissinger et al., 2017; Cui et al., 2018; Raffaelli et al., 2020; Schaefer and McLeish, 2022), remains to couple the (continuum-time) conformational dynamics to model the stochastic (instantaneous) association and dissociation of reversible bonds between the molecules. To circumvent the current methodological challenges, in the present study, we will employ the fully stochastic Bond-Fluctuation Model (Carmesin and Kremer, 1988, 1990), which by modeling the molecular dynamics on a 3D lattice successfully describes the (self-avoiding) random walk statistic of flexible and persistent chains (Bates, 2002; Feric et al., 2016; Harmon et al., 2017), LLPS, Reister et al. (2001), Reister and Müller (2003), ring-polymers (Subramanian and Shanbhag, 2009), and cross-linking reactions (Trautenberg et al., 1995). Crucially, as the dynamics are fully stochastic and discrete, they can be addressed using previously developed kinetic Monte Carlo (kMC) schemes (Lukkien et al., 1998).

In the following, we will present a simple sticker-and-spacer model for the dimerisation and (linear) oligomerisation of unfolded Aβ, where the cooperativity of growth is determined both by the chain conformation (i.e., intrinsic disorder) and by the binding energies for dimerisation/nucleation, ε_n_, and elongation, ε_e_, and where hydrophobic interactions are modeled using a short-ranged interaction energy, ε_H_. Subsequently, we will present the bond-fluctuation model (BFM) using which we simulate the aggregation of the Aβ. In our analysis, we first investigate how the hydrophobic interactions affect the partial collapse of the molecule, and how it may lead to LLPS. We then show that these interactions facilitate dimerisation, as well as the further cooperative growth into longer oligomers. Finally, we demonstrate that increasing Aβ_1−42_ relative to Aβ_1−40_ promotes aggregation.

## 2. Theory and method

### 2.1. Introduction: Equilibrium statistics of self-assembly

**Figure 1:**
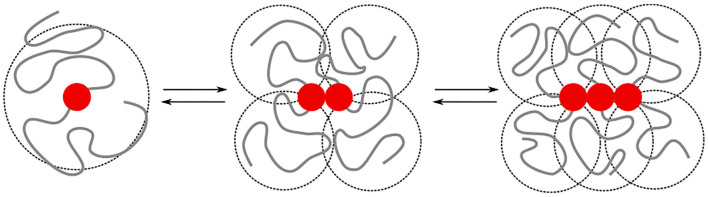
We model the growth of an oligomer through reversible bonds between bivalent stickers (red bead). The cooperativity is controlled by a difference in the binding energy for dimerisation/nucleation (ε_n_) and elongation (ε_e_). The total binding energy is further affected by repulsive excluded volume interactions between beads, as well as attractive hydrophobic interactions (ε_H_) between beads within a short interaction range. The excluded volume interactions and hydrophobic interactions depend on the overlap (indicated by the dashed circles) of the intrinsically disordered sequences; the amount of overlap non-linearly increases with the length of the chains. In this study, we study how the added hydrophobic interactions and increased chain length of Aβ_1−42_ compared to Aβ_1−40_ affects the self-assembly into oligomers.

The irreversible formation of Aβ aggregates is nucleated by oligomers, which themselves are reversible and on most occasions re-dissolve into Aβ monomers (Michaels et al., 2020). The formation of oligomers can on a coarse-grained scale, be described by chemical-reaction equations of the form A_1_⇌A_*n*_, where *n* > 1 is the order of the reaction. This reaction order gives a measure for the size of the oligomers but does not provide any structural information [e.g., whether the oligomers are amorphous clusters, rings, or linear chains, and if there are parallel or anti-parallel assemblies of poly-peptide sequences (Miller et al., 2010)]. While this structural information is typically studied at the level of single amino acids and atoms, the observations on the intrinsic disorder in Aβ through its random-coil conformations through both circular dichroism (Danielsson et al., 2005) and nuclear magnetic resonance spectroscopy (Wälti et al., 2015; Roche et al., 2016), begs the question of to what extent concepts from coarse-grained polymer physics can be used to understand the difference in self-assembly behavior of Aβ_1−40_ and Aβ_1−42_. The key idea that we explore is that the specific binding between substrands of the chain leads to interactions between the intrinsically disordered parts of the protein. These include both repulsive excluded-volume interactions and attractive hydrophobic interactions, (Figure 1); their contributions grow non-linearly with the size of the oligomer.

To investigate these ideas, we will describe both species as a randomly coiled polymer that is composed of hydrophilic and hydrophobic beads, as well as one ‘sticker' bead. This sticker bead forms reversible intermolecular bonds and leads to the formation of an oligomer. In general, any two oligomers of size *m* ≥ 1 and *n* ≥ 1 may reversibly assemble into an aggregate of size *m* + *n* through the chemical equilibrium equation (Martin, 1996; van der Schoot, 2005; de Greef et al., 2009).


(1) $$\begin{array}{l} A_{m}+{\text{A}}_{n}⇌{\text{A}}_{m+n}. \end{array}$$

The equilibrium constants of the reactions are non-universal and may be different for each oligomerisation step due to the formation of rings (Cates and Candau, 1990) or due to detailed phenomena, such as the parallel and anti-parallel assembly of poly-peptides. These different mechanisms may be summarised into generic schemes of (anti-)cooperative growth, where in the anti-cooperative case the nucleus is stable and only grows into larger species at high chemical potentials, whereas in the cooperative case, the nucleus is unstable and, once formed, either re-dissolves or rapidly grows into larger (stable) species. As the exact nature of the Aβ species is still under debate, we here represent these overall ideas by a simple ‘dimerisation-and-elongation' model (de Greef et al., 2009; Kulkarni et al., 2017), where we have a precise *in silico* control over the cooperativity of growth. We discuss this binding mechanism of the stickers in detail in Section 2.5. First, we will discuss the parametrisation of the protein as a randomly colloid polymer, discuss the Bond-Fluctuation model by which we simulate its equilibrium statistics, and the simple hydrophobicity model.

### 2.2. Parametrisation of unfolded Aβ

**Figure 2:**
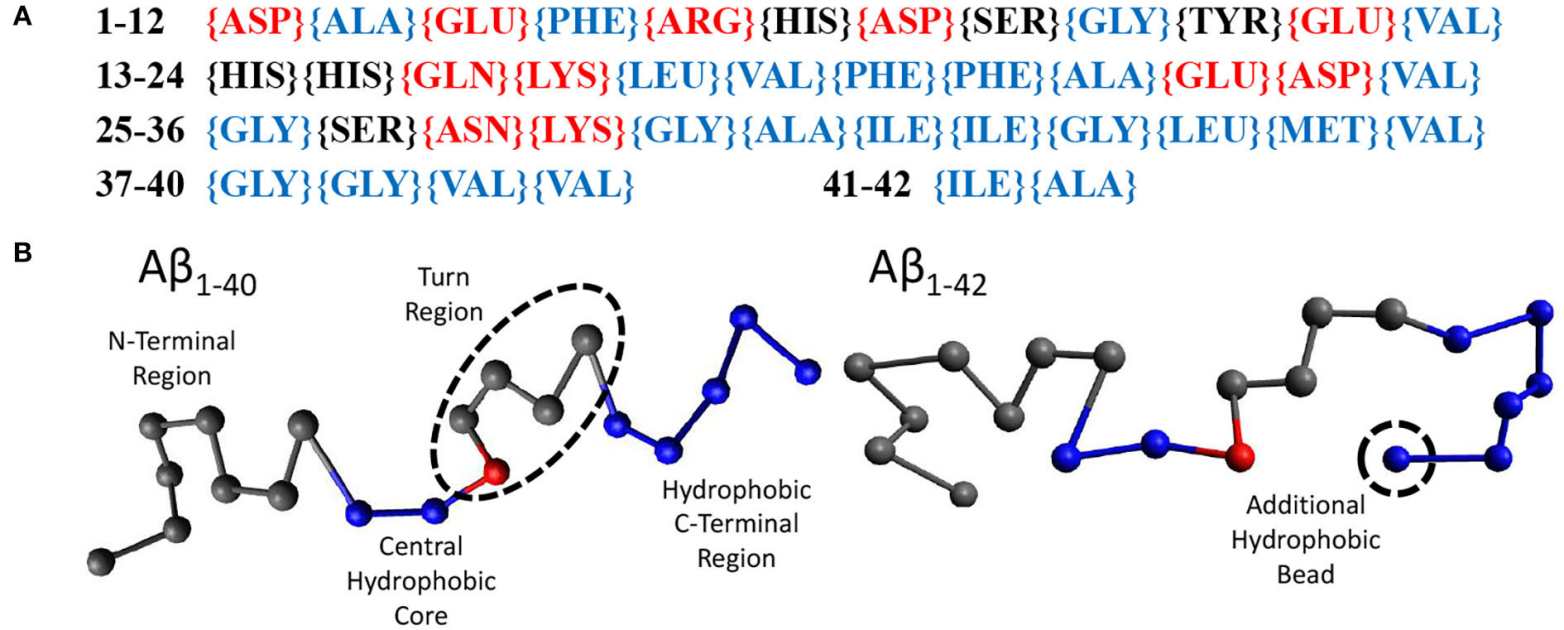
**(A)** The amino acid sequence of Aβ_1−42_. Amino acids labeled red are hydrophilic, black are neutral, and blue are hydrophobic. **(B)** Our beads on a string model for both Aβ_1−40_ and Aβ_1−42_ with the N-terminus to the C-terminus going from left to right. Each bead represents 2 amino acids. Gray beads are inert, representing the N-terminus (1–16) and the turn region (24–27). Blue beads are hydrophobic, representing the central hydrophobic core (17–21), the second hydrophobic region (29–35) and the hydrophobic C-terminus (36–40/42). Red beads are capable of dimerising with other red beads.

Random walk statistics of polymers typically emerge when the contour length is more than 10 times its persistence length and can be modeled using the ‘Bond-Fluctuation Model' (refer to next Section). The smallest length scale of this model is the persistence length which for polypeptides is one or a few amino acids. Using these considerations, we have chosen to use 20 beads to model Aβ_1−40_ and 21 beads to model Aβ_1−42_. Within our model, a bead may either be hydrophilic, hydrophobic, or may be a sticker. We have parameterised this model using the sequence of amino acids in Aβ_1−42_, (Figure 2A), which displays the hydrophilic residues in red, the neutral ones in black, and the hydrophobic ones in blue. Our model for Aβ (Figure 2B) was based on the structure of Aβ_1−42_ in Zhu et al. (2012). The first eight gray beads represent the first 16 amino acids corresponding to the hydrophilic N-terminal region. The central hydrophobic core is modeled as two blue (hydrophobic) beads representing amino acids 17-21. A red ‘sticky' bead which is capable of dimerising with other red beads was added into the turn region to recapitulate the intra- and inter-chain salt bridges that can occur in this region. The remaining hydrophobic beads represent the C-terminal region with Aβ_1−42_ containing one extra bead to account for the addition of two hydrophobic amino acids. In principle, the properties of the beads may be informed from MD simulations or structural information. However, at this high level of coarse graining the exact sequence is not expected to qualitatively alter the conclusions of our study, in which we have only observed the formation of linear oligomers (i.e., the stickers bind into supramolecular chains) and droplets, but in which no higher-order self-assembled structures such as micelles or membranes were formed (see Results). These may be expected if the hydrophobic/hydrophilic beads would have been arranged into a co-block configuration, and/or if the stickers would have been placed at the chain ends.

### 2.3. Method: Bond-fluctuation model

We model the chain conformations by placing the polymer segments on the sites of a discrete lattice, following the so-called Bond-Fluctuation Model (BFM) (Carmesin and Kremer, 1988, 1990). This approach originates from the success of lattice models to predict phenomena such as LLPS, including in conditions near the critical point where the correlation-length diverges and mean-field models break down (Hohenberg and Halperin, 1977; Schaefer, 2018; Schaefer et al., 2019). Similarly, the lattice models have provided computationally efficient means to sample the configuration space of polymers. Early lattice models for polymers place polymer segments on a single lattice site (Binder, 1987) but underestimated the Rouse dynamics of the chains due to kinetically trapped states. This was remedied in the BFM by letting a segment occupy 8 sites on a 3D cubic lattice (4 sites on a 2D square lattice) and where the bond lengths can fluctuate from 2 to $\sqrt{10}$ in lattice units (Carmesin and Kremer, 1988, 1990). This approach was proven competitive with off-lattice models both in terms of computational convenience and in physical accuracy in (and beyond) the examples mentioned in the Introduction (Trautenberg et al., 1995; Reister et al., 2001; Bates, 2002; Reister and Müller, 2003; Subramanian and Shanbhag, 2009; Choi et al., 2019).

Within the BFM, the polymer conformations and/or dynamics are modeled using kinetic Monte Carlo (kMC) time steps in which a monomer may move in 6 directions on the lattice if this does not (1) overstretch or understretch the bond between two monomers within the chain or (2) lead to double-occupied lattice sites. This leads to a list of *N*_enabled_ ≤ 6*N*_monomers_ possible processes that may occur during the time step, of which only one (or none) may take place. Which process, *i*, may take place is selected using the rate,


(2) $$\nu_{i}=\left\{ \begin{array}{l} \nu_{0},\text{ifΔ}E_{\text{i}}\leq 0\\ \nu_{0}\exp(\text{−Δ}E_{\text{i}}/kT),\text{ifΔ}E_{\text{i}}>0, \end{array}\right.$$

where ν_0_ is an elementary rate (typically of the order 1−100μs^−1^) and where Δ*E*_i_ is the change in energy. In the algorithm that we use, a process is randomly selected out of the *N*_enabled_ list of processes. The process is then executed if the system energy is unchanged or decreases Δ*E*_i_ ≤ 0. However, if the energy would increase, the process is only executed with a probability *p* = exp(−Δ*E*_i_/*kT*) and rejected otherwise; this decision is carried out using random numbers drawn using a SIMD-oriented Fast Mersenne Twister (Saito and Matsumoto, 2008). Regardless, if the process is accepted or rejected, the time is increased with Δ*t* = 1/(*N*_enabled_ν_0_). The computational efficiency of the method relies on the fact that following a Monte Carlo step during which monomer moves, only the rates of monomers in the vicinity of this monomer need to be recalculated (Lukkien et al., 1998).

### 2.4. Parametrisation: Hydrophobicity of spacers

**Figure 3:**
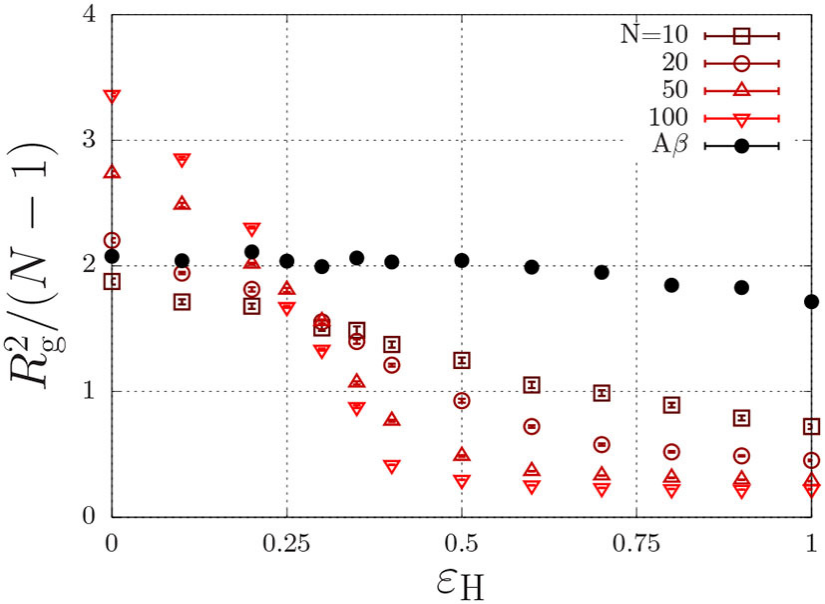
Polymer size, $R_{\text{g}}^{2}/\left(N-1\right)$, (*R*_g_ is the radius of gyration in units of the lattice spacing) as a function of the hydrophobic interaction energy ε_H_/*k*_B_*T* for a various number of beads per chain, *N*. The Aβ model has a total of *N* = 20 beads, of which 7 are hydrophobic.

To model the attraction between two hydrophobic monomers we follow the approach by Reister et al., and use a square-well potential (Reister et al., 2001).


(3) $$U(r)=\{\begin{array}{l} -\varepsilon_{\text{H}},\text{ for }r\leq \sqrt{10},\\ 0,\text{ otherwise}, \end{array}$$

where *r* is the distance (in units of the lattice spacing) between the two monomers, and where ε_H_ describes non-specific (e.g., hydrophobic) interactions (Reister et al., 2001). This interaction energy enables the parametrisation of intrinsically disordered poly-peptides through the radius of gyration, and may in principle depend on conditions such the temperature and the ionic strength (Müller-Späth et al., 2010; Wuttke et al., 2014). This parametrisation is done using the Flory exponent υ, which describes the swelling of a polymer through the radius of gyration as $R_{\text{g}}=lN^{\upsilon }/\sqrt{6}$, with *l* the step length between segments. In good solvent conditions, the chain is swollen due to intramolecular self-excluded volume interactions and υ = 0.588. Completely insoluble chains, described with a large value of ε_H_, collapse to a compact sphere with υ = 1/3. At θ conditions, the hydrophobic interactions exactly cancel the excluded-volume interactions and the chain obeys random-walk statistics, which are characterized by υ = 1/2. To find the θ-condition, we measure the radius of gyration for chains with various chain length, *N*, as a function of ε_H_ (Paul et al., 2005; Steinhauser, 2005). As $R_{\text{g}}^{2}/N$ is independent of the chain length at the theta condition, the ε_H_ value at which all curves intersect represents the θ condition. From Figure 3, we find that this occurs at ε_H_/*k*_B_*T* ≈ 0.27 for (homopolymer) chains with identical subunits.

As discussed in the previous section, our model for Aβ describes the protein as a copolymer with both hydrophilic and hydrophobic units. The solid circles in Figure 3 show that the radius of gyration, $R_{\text{g}}=lN^{\upsilon }/\sqrt{6}$ of this model polypeptide is relatively insensitive to the hydrophobic interaction parameter. To estimate the overlap concentration above which the intramolecular excluded-volume interactions are screened, we use the end-to-end distance, given by $R_{\text{e}}=lN^{\upsilon }\approx 3.3$ nm, where Aβ has *N* = 40 amino acids, and where *l* = 0.36 nm is the typical step length of amino acids (Müller-Späth et al., 2010; Wuttke et al., 2014). Using the molecular volume $V_{\text{m}}=\left(4/3\right)\pi R_{\text{e}}^{3}$, we find an overlap concentration of c_*_ = *M*_w_/(*N*_A_*V*_m_) ≈ 100 mg/ml = 0.025 M, where we used *M*_w_ = 4.5 kg/mol. In typical experiments, aggregation is observed well below the overlap concentration (e.g., Aβ_1−40_ aggregates below 1 mg/ml Hortschansky et al., 2005 and Aβ_1−42_ aggregates at concentrations as low as 90 nM Novo et al., 2018).

While those experimental conditions may suggest dilute conditions in which excluded-volume interactions are unimportant, inside LLPS condensates the concentration is signicantly higher, and may in fact exceed the overlap concentration. To identify LLPS in our simulations, we focus on structural coarsening phenomena such as Ostwald or Lifshitz-Slyozov-Wagner (LSW) ripening and/or Brownian coalescence (Bray, 2002). The dynamics of coarsening are typically associated with a characteristic length scale (e.g., the radius of a droplet) that grows with the one-third power of time as R^*^−*R*^0^ ∝ *t*^1/3^ with *R*_0_ the initial length scale, which may emerge through nucleation or spinodal decomposition. In our simulations, we determine R* using the following recipe (Singh et al., 2012; Schaefer, 2018; Schaefer et al., 2019): For a given time, *t*, we calculate the order-parameter field ψ(**r**_*i*_), with **r**_*i*_ the spatial coordinate of a lattice site with *i* the index of a lattice site. The value of ψ(**r**_*i*_) is set to 1 if the site is occupied by a hydrophobic monomer, and to −1 otherwise. We then calculate the 3D Fourier transform $\hat{\psi }\left(\text{q}\right)$ and obtain the structure factor $S\left(q\right)=\langle |\hat{\psi }\left(\text{q}\right){|}^{2}\rangle$, where 〈.〉 is the spherical/angular average. Next, we obtain the spatial correlation function *C*(*R*) as the inverse Fourier transform of *S*(*q*). As discussed previously (Singh et al., 2012; Schaefer, 2018; Schaefer et al., 2019), numerically robust measures for the characteristic length scale R^*^ are the first root (i.e., given by *C*(R^*^) = 0) and the first minimum for which *C*(R^*^) < 0 and *C*′(R^*^) = 0. In our analysis, we will use both measures to assess if structural coarsening occurs in our simulations.

### 2.5. Parametrisation: Binding of stickers

As motivated in Section 2.1, we will model oligomerisation through the reversible binding of divalent stickers through a ‘dimerisation-and-elongation' mechanism, where each sticker can either form a single bond or two bonds with other stickers. The dimerisation reaction is


(4) $$\begin{array}{l} 2{\text{A}}_{1}\overset{K_{\text{n}}}{⇌}{\text{A}}_{2}, \end{array}$$

where


(5) $$K_{\text{n}}=\frac{1}{\upsilon }\exp\left(-\text{Δ}H_{\text{n}}/k_{\text{B}}T+\text{Δ}S_{\text{n}}/k_{\text{B}}\right),$$

is the equilibrium constant for nucleation, with υ the characteristic volume, Δ*H* < 0 is the binding enthalpy and with Δ*S*_n_ > 0 the entropic penalty of dimerisation. In this equation, Δ*H*_n_ is controlled by the binding energy ε_n_ > 0 and the hydrophobic interaction strength ε_H_. In the absence of hydrophobic interactions, Δ*H*_n_ = −ε_n_ is exact. The entropic penalty essentially originates from an excluded-volume interaction due to a limitation of the internal degrees of freedom (DOF) of two chains that undergo dimerisation (refer to Section 3.1). In that section, we will show that these internal DOF are affected by the hydrophobicity (an increased hydrophobicity partially collapses the chain and limits the DOF prior to dimerisation), and by the concentration (above the overlap concentration the free chains have fewer DOF prior to dimerisation), such that an increasing hydrophobicity and concentration reduce the entropic penalty and enhance dimerisation.

For subsequent oligomerisation steps, i.e., for *n* > 1, we describe the equilibrium statistics using


(6) $$\begin{array}{l} A_{1}+{\text{A}}_{n}\overset{K_{\text{e}}}{⇌}{\text{A}}_{n+1} \end{array}$$

with


(7) $$K_{\text{e}}=\frac{1}{\upsilon }\exp\left(-\text{Δ}H_{\text{e}}/k_{\text{B}}T+\text{Δ}S_{\text{e}}/k_{\text{B}}\right),$$

in which Δ*H*_e_/*k*_B_s*T* = −ε_n_+*U*_hydrophobic_+*U*(θ) is composed of an oligomeration/elongation energy, ε_n_ > 0, and hydrophobic interactions as before. The free energy of binding is modeled as completely entropic using a square-well bending potential (BFM simulations with smoother potentials were previously discussed in the literature, refer to Weber et al., 1999; Bates, 2002; Paul et al., 2005).


(8) $$U(\theta )=\{\begin{array}{l} \infty ,\text{ for }|\theta -\pi |\leq \theta_{\text{max}},\\ 0,\text{ otherwise, } \end{array}$$

for the angle, θ, between the intermolecular bonds between stickers. Here, θ = π represents fully extended bonds. Hence, for small values of θ_max_, the persistence length increases and the chain of stickers approaches a rigid rod. In our simulations, we set θ_max_ = π/18 (equivalent to 10 degrees), which ensures sufficient rigidity to avoid ring formation but sufficient flexibility to avoid lattice artifacts.

One of the key predictions of our simulations will be the dependence of the fraction of aggregated material on the concentration and hydrophobicity. We will interpret these findings using analytical predictions in the limit where hydrophobic interactions are absent and where the entropic penalties are constant. In this limit, there exist some known analytical predictions (van der Schoot, 2005). The starting point to obtain these is by writing the equilibrium constants as $K_{\text{n}}=\left[{\text{A}}_{2}\right]/{\left[{\text{A}}_{1}\right]}^{2}$ and as *K*_e_ = [A_*n*+1_]/[A_*n*_][A_1_], which are constant for all *n* ≥ 2, so that the concentration of any aggregate [A_*n*_] with *n* ≥ 2, can be expressed in terms of the concentration of unbound Aβ, [A_1_], as


(9) $$K_{\text{e}}[{\text{A}}_{n}]=\sigma {\left(K_{\text{e}}[{\text{A}}_{1}]\right)}^{n},$$

with σ ≡ *K*_n_/*K*_e_ the so-called cooperativity factor.

The concentrations of unbound Aβ is obtained from the mass balance,


(10) $$\begin{array}{l} \rho =\sum_{n=1}^{\infty }n[A_{n}]\Rightarrow K_{\text{e}}\rho =K_{\text{e}}[{\text{A}}_{1}]+\sigma \sum_{n=2}n{\left(K_{\text{e}}[{\text{A}}_{1}]\right)}^{n}\\ =\sigma \frac{K_{\text{e}}[{\text{A}}_{1}]}{{\left(1-K_{\text{e}}[{\text{A}}_{1}]\right)}^{2}}, \end{array}$$

where ρ is the (experimentally-controlled) overall concentration of Aβ. Using the standard sum $\overset{\infty }{\underset{n=1}{\sum }}nx^{n}=x/{\left(1-x\right)}^{2}$ for |*x*| < 1 and the fraction of aggregated molecules, *f* ≡ 1−[A_1_]/ρ, this mass balance can be written as Martin (1996) and de Greef et al. (2009).


(11) $$0=-f+\sigma \frac{K_{\text{e}}\rho {\left[1-f\right]}^{2}(2-K_{\text{e}}\rho [1-f])}{{\left(1-K_{\text{e}}\rho [1-f]\right)}^{2}},$$

which provides an implicit dependence of *f* on ρ.

This equation has three asymptotic limits of interest, namely the strongly anti-cooperative case where dimers do not grow into larger aggregates, *K*_e_ → 0 (resulting in σ → ∞) leads to Martin (1996).


(12) $$\begin{array}{l} f=1-\frac{-1+\sqrt{1+8K_{\text{n}}\rho }}{4K_{\text{n}}\rho }. \end{array}$$

In the non-cooperative case, or “isodesmic” case (Smulders et al., 2010), *K* ≡ *K*_e_ = *K*_n_ (Martin, 1996),


(13) $$f=1-\frac{1+2K\rho -\sqrt{1+4K\rho )}}{2{\left(K\rho \right)}^{2}},$$

and in the strongly cooperative case, σ → 0 (van der Schoot, 2005; Smulders et al., 2010), we have *f* = 0 for *K*_e_ρ < 0 and


(14) $$\begin{array}{l} f=1-\frac{1}{K_{\text{e}}\rho }, \end{array}$$

for *K*_e_ρ ≥ 0. These equations show that from the anti-cooperative to the cooperative case, the transition from unbound to aggregated material becomes increasingly sharp. In the following, we will discuss the influence of hydrophobic and excluded-volume interactions on self-assembly in terms of the elongation constant *K*_e_ and the cooperativity factor, σ.

## 3. Results and discussion

### 3.1. Dimerisation and LLPS

**Figure 4:**
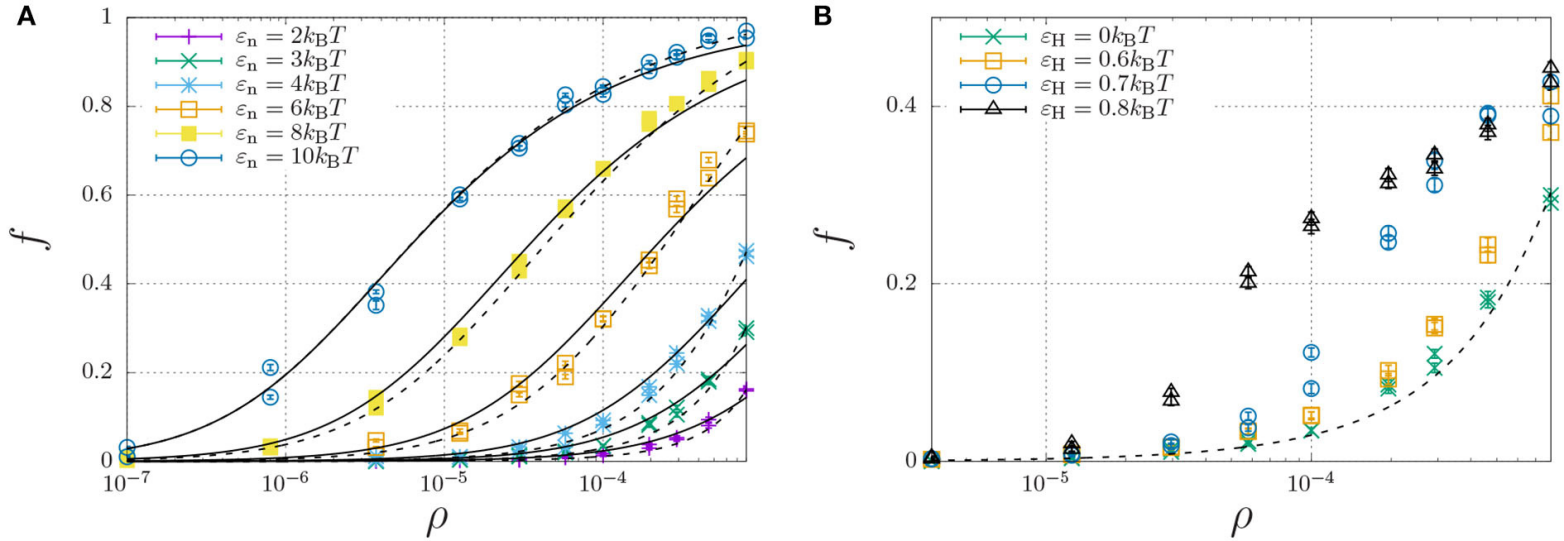
Fraction of dimerised Aβ, *f*, plotted against the number density, ρ (in units of the lattice spacing). Well below the overlap concentration of ρ = 10^−4^ (corresponds roughly to 100 mg/ml, refer to main text) the solution can be considered dilute, while at higher concentrations excluded volume interactions become important. **(A)** The dimerisation energy ε_n_ is varied from 2 to 10*k*_B_*T* without hydrophobic interactions (ε_H_ = 0). The solid curves are individual fits using Equation (12) with a dimerisation constant *K*_n_ = *K*_n,0_ exp(ε_n_) with a non-constant *K*_n,0_ (Table 1), while the dashed curves represent a simultaneous fit of all data using *K*_n,0_ = *K*_n,00_ exp[(∂[Δ*S*_n_/*k*_B_]/∂ρ)ρ] with constant *K*_00_ = 6.85 and an entropic excluded-volume correction $\left(\partial \left[\Delta S_{\text{n}}/k\text{B}\right]/\partial \rho \right)=1.3\cdot 10^{3}$. **(B)** The dimerisation energy is fixed ε_n_ = 3*k*_B_*T*, while the hydrophobicity is increased from 0 up to ε_H_ = 0.8. The dashed and dotted curve are calculated using *K*_n_ = 6.85 exp(3+1300ρ) and *K*_n_ = 6.85 exp(3.6+1300ρ), respectively. The data for ε_H_ = 0.7 and 0.8 are underestimates, as ongoing LLPS enables the slow increase of aggregate sizes.

To investigate how hydrophobic and steric interactions affect dimerisation, we have compared the self-assembly of chains with such interactions to the completely hydrophilic counterpart with ε_H_ = 0, and disabled oligomerisation (i.e., ε_e_ = 0). We have then simulated the molecular self-assembly of 100 chains in a periodic simulation box with sizes ranging from 50 × 50 × 50 to 1,000 × 1,000 × 1,000, with dimerisation energies ranging from ε_n_ = 2 to 10*k*_B_*T*. The resulting fraction of dimerised chains, *f*, against the number density ρ (in the number of molecules per box size), is represented by the symbols in Figure 4A.

We have curve fitted each data set with fixed dimerisation energy, ε_e_, using the dimerisation model of Equation (12) (solid curves) with the equilibrium constant *K*_n_ = *K*_n,0_ exp(ε_n_). While *K*_n,0_ is explicitly independent of ε_n_, the curve fits yielded the apparent dependence ln *K*_n,0_ ≈ 3.0 − 1.1ε_n_ (Table 1). This is caused by the fact that the dimerization concentration (characterized by the inflection point of the self-assembly curve) shifts to higher concentrations (above the overlap concentration of ρ ≈ 10^−4^, see Section 2.4) for decreasing interaction strengths. Following Flory's approach to self-avoiding walk statistics, we modify the entropy for excluded-volume interactions using a mean-field description, Δ*S*_n_ = Δ*S*_n,0_+(∂Δ*S*_n_/∂ρ)ρ, and write *K*_n_ = *K*_n,00_ exp(ε_n_+(∂[Δ*S*_n_/*k*_B_]/∂ρ)ρ. Using this modification, we have been able to capture all dimerisation curves in Figure 4A with fixed *K*_00_ = 6.85 and excluded-volume correction $\left(\partial \left[\Delta S_{\text{n}}/k\text{B}\right]/\partial \rho \right)=1.3\cdot 10^{3}$ (dashed curves). As expected, this correction is insignificant below the overlap concentration, ρ < 10^−4^, but significantly affects the self-assembly curves at higher concentrations.

**Table 1:** Values for the equilibrium constant *K*_n,0_ determined by curve-fitting Equation 12 to the simulated data for various values of the dimerisation energy ε_n_.

**ε_n_/*k*_B_*T***	***K*_n, 0_**
2	16.63 ± 0.73
3	15.10 ± 0.71
4	13.48 ± 0.82
6	10.61 ± 0.77
8	9.12 ± 0.45
10	6.85 ± 0.37

The curve-fits are shown as solid lines in Figure 4. A lin-log regression yields an *apparent* relationship ln *K*_n,0_ ≈ 3.0 − 1.1ε_n_/*k*_B_*T* (refer to main text).

We now focus our attention on the dimerisation curve with ε_n_ = 3*k*_B_*T*, which has a very low fraction of dimers below the overlap concentration but a finite fraction of dimers at higher concentrations, and increase the hydrophobic-interaction parameter, ε_H_, from 0 to 0.8*k*_B_*T* (Figure 4B). We find that up to a value of 0.6*k*_B_*T*, the hydrophobicity appears to only modestly modify the equilibrium constant as *K*_n_ ≈ 6.85 exp(ε_n_ + ε_H_ + 1300ρ); we speculate that only the central hydrophobic beads contribute to the enhancement of dimerisation. For stronger hydrophobic interactions, however, the shape of the self-assembly curve can no longer be described using the simple dimerisation model.

**Figure 5:**
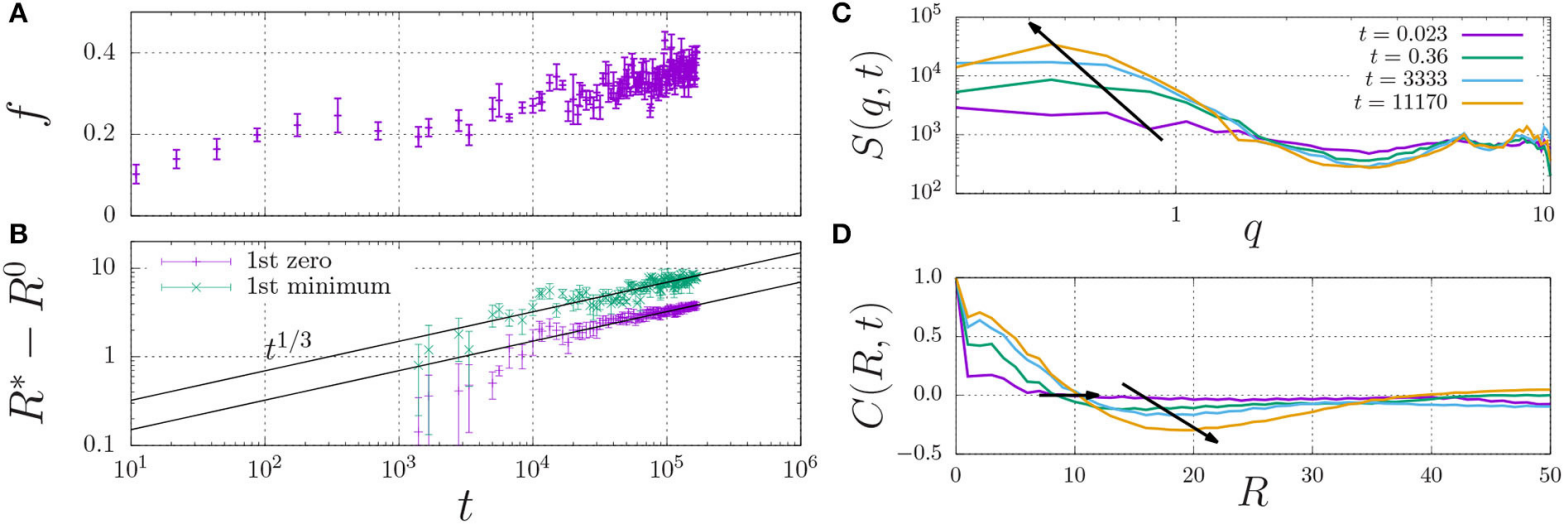
Fraction of dimerised material, *f*, **(A)** and characteristic length scale, R^*^−*R*^0^ **(B)** against time. The characteristic length scale, R^*^ is determined by i) calculating the dynamic structure factor, *S*(*q*) **(C)**, ii) taking the inverse Fourier transform to obtain the radial correlation function, *C*(*R, t*) **(D)**, and iii) determining its first root *C* = 0 (here, we use the initial offset *R*^0^ = 8) and first minimum for which *C* < 0 (here, *R*^0^ = 12). The arrows indicate the time dependence of the dominant wavenumber *q* **(C)** and the two measures for R^*^ **(D)**.

In fact, the datapoint for ε_H_ ≥ 0.7 in Figure 4B is not fully converged, and the fraction of dimers increases in a slow process, as indicated in Figure 5A. This figure shows that the fraction of dimers, *f*, reaches a plateau at *f* ≈ 0.2 for times *t* > 100 up to *t* ≈ 3, 000, but then slowly increases up to *f* ≈ 0.4 at *t* = 10^5^ without any sign of convergence to a higher plateau value. This process is a consequence of the slow formation of large structures, as indicated by the typical length scales in the system Figure 5B, which increase as R^*^−*R*^0^ ∝ *t*^1/3^, which is a hallmark of LLPS, refer to Section 2.4. Here, we determined R^*^ using the structure factor *S*(*q, t*) (top right), and its corresponding spatial correlation function, *C*(*R, t*), (bottom right). We quantified R^*^ using the first root (*C*(R^*^, *t*) = 0, *R*^0^ = 8) and the first minimum (*C*(R_*_, *t*) < 0 and ∂*C*/∂*R* = 0, *R*^0^ = 12).

These findings indicate that sufficiently strong hydrophobic interactions may lead to the formation of droplets through LLPS, which inside the droplets increases the concentration of dimerising units beyond the overlap concentration (which screens the excluded-volume interactions), and provides the mass action needed to induce dimerisation.

### 3.2. Oligomerisation

Now that we have investigated the dimerisation of Aβ, we will investigate the growth of larger oligomers. For that purpose, we again first focus on the case without any hydrophobic interactions, as this enables us to isolate the impact of excluded-volume interactions on the (anti-)cooperativity of self-assembly. This also enables us to select nucleation and elongation energies of interest in the subsequent simulations, in which we do switch on hydrophobic interactions. Akin to the dimerisation case, we have simulated the self-assembly of 100 Aβ_1−40_ chains in box sizes ranging from 36 × 36 × 36 to 500 × 500 × 500. In these simulations, we have switched off the non-specific/hydrophobic interactions ε_H_ = 0 and varied the nucleation energies in the range ε_n_ = 1 − 9*k*_B_*T* and the elongation energies in the range ε_e_ = 8 − 14*k*_B_*T*.

The simulations yielded the fraction of aggregated material, *f*, as a function of the concentration, to which we have curve fitted the theoretical model of **Equation (11)**. From the curve-fits, we have extracted the cooperativity factor σ and the equilibrium constant *K*_e,0_ as a function of the dimerisation energy. Here, *K*_e,0_ is defined by


(15) $$K_{\text{e}}=\upsilon^{-1}\exp(-\text{Δ}H_{\text{e}}+\text{Δ}S_{\text{e}}/k_{\text{B}})\equiv K_{\text{e},0}\exp(\varepsilon_{\text{e}}/k_{\text{B}}T).$$

**Figure 6:**
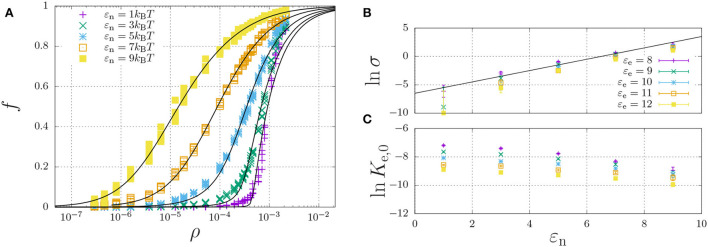
Self-assembly of a hydrophilic chain (ε_H_ = 0) for a fixed elongation energy ε_e_ = 9*k*_B_*T* and varying dimerisation energy ε_n_. **(A)** The fraction of aggregated material is plotted against the concentration. For each value of ε_n_, the data was curve-fitted using Equation (11) with cooperativity factor σ **(B)** and the equilibrium constant *K*_e_ = *K*_e,0_ exp(ε_e_) **(C)** as fitting parameters. For **(B,C)**, each point represents the mean and standard error of 5 simulations.

The results are tabulated in Table 2 and plotted in Figures 6B,C (discussed below).

**Table 2:** Shown are the results from varying ε_n_ for multiple different values of ε_e_, in the absence of hydrophobic interactions i.e., ε_H_ = 0*k*_B_*T*.

**ε_n_/*k*_B_*T***	**ε_e_/*k*_B_*T***	**σ**	***K*_n,0_**
1	8	2.15 × 10^−3^±2.31 × 10^−3^	2.24 × 10^−4^±1.08 × 10^−5^
3	8	5.41 × 10^−2^±1.24 × 10^−2^	1.82 × 10^−4^±8.96 × 10^−6^
5	8	3.91 × 10^−1^±3.73 × 10^−2^	1.26 × 10^−4^±5.83 × 10^−6^
7	8	1.90 ± 1.65 × 10^−1^	7.29 × 10^−5^±4.43 × 10^−6^
9	8	8.15 ± 2.10	3.87 × 10^−5^±8.80 × 10^−6^
1	9	1.33 × 10^−4^±4.68 × 10^−4^	3.87 × 10^−4^±1.89 × 10^−5^
3	9	2.10 × 10^−2^±9.29 × 10^−3^	3.25 × 10^−4^±1.72 × 10^−5^
5	9	2.34 × 10^−1^±3.38 × 10^−2^	2.38 × 10^−4^±1.31 × 10^−5^
7	9	1.50 ± 1.54 × 10^−1^	1.61 × 10^−4^±1.11 × 10^−5^
9	9	5.55 ± 1.54	7.92 × 10^−5^±1.86 × 10^−5^
1	10	1.00 × 10^−5^±1.54 × 10^−4^	6.89 × 10^−4^±3.19 × 10^−5^
3	10	5.34 × 10^−3^±3.74 × 10^−3^	5.83 × 10^−4^±2.60 × 10^−5^
5	10	1.53 × 10^−1^±3.04 × 10^−2^	4.49 × 10^−4^±2.87 × 10^−5^
7	10	1.08 ± 1.36 × 10^−1^	3.26 × 10^−4^±2.55 × 10^−5^
9	10	6.02 ± 1.43	2.10 × 10^−4^±4.26 × 10^−5^
1	11	1.00 × 10^−5^±7.55 × 10^−5^	1.15 × 10^−3^±3.72 × 10^−5^
3	11	9.06 × 10^−3^±5.57 × 10^−3^	1.05 × 10^−3^±5.99 × 10^−5^
5	11	8.19 × 10^−2^±2.17 × 10^−2^	7.98 × 10^−4^±5.03 × 10^−5^
7	11	8.85 × 10^−1^±1.15 × 10^−1^	6.62 × 10^−4^±5.07 × 10^−5^
9	11	4.60 ± 1.18	4.64 × 10^−4^±9.82 × 10^−5^
1	12	4.58 × 10^−5^±1.79 × 10^−4^	2.23 × 10^−3^±7.59 × 10^−5^
3	12	3.77 × 10^−3^±3.18 × 10^−3^	1.83 × 10^−3^±8.20 × 10^−5^
5	12	8.19 × 10^−2^±1.61 × 10^−2^	1.51 × 10^−3^±6.71 × 10^−5^
7	12	5.94 × 10^−1^±9.53 × 10^−2^	1.19 × 10^−3^±9.93 × 10^−5^
9	12	2.88 ± 5.63 × 10^−1^	7.77 × 10^−4^±1.15 × 10^−4^
1	14	5.95 × 10^−3^±5.23 × 10^−3^	8.12 × 10^−3^±5.12 × 10^−4^
3	14	1.65 × 10^−2^±1.14 × 10^−2^	7.99 × 10^−3^±6.79 × 10^−4^
5	14	7.83 × 10^−2^±2.53 × 10^−2^	4.97 × 10^−3^±4.04 × 10^−4^
7	14	3.26 × 10^−1^±6.66 × 10^−2^	3.43 × 10^−3^±3.03 × 10^−4^
9	14	7.21 × 10^−1^±1.36 × 10^−1^	2.00 × 10^−3^±2.07 × 10^−4^

Each simulation used 100 Aβ_1−40_ chains in box sizes ranging from 36 x 36 x 36 to 500 x 500 x 500. Each value of σ and *K*_n,0_ represents the mean and standard error from 3 separate simulations.

A representative self-assembly curve, obtained for fixed ε_e_ = 9*k*_B_*T*, is shown in Figure 6A. This panel shows the fraction of aggregated material, *f*, increases with an increasing number density, ρ. The sigmoidal curve becomes increasingly sharp with decreasing dimerisation energy, which, as expected (refer to Section 2.5), indicates increasing cooperativity of self-assembly. Indeed, Figure 6B shows that the logarithm of the cooperativity factor increases linearly with increasing dimerisation energy, as expected. On the other hand, the elongation constant *K*_e,0_ is in principle expected to be independent of the dimerisation energy; however, Figure 6C shows it apparently decreases with increasing dimerisation energy. We attribute this apparent dependence to the excluded-volume interactions becoming more present at higher concentrations, as we found above in the dimerisation curves of Figure 4.

To investigate the effect of non-specific hydrophobic interaction on oligomerisation, we have used the parameters ε_n_ = 3*k*_B_*T* and ε_e_ = 9*k*_B_*T* of a hydrophilic chain that cooperatively self-assembles at reasonably high concentrations. This ensured that low-concentration structuring due to hydrophobic interactions would not necessitate larger (and computationally expensive) box sizes. We have then used the sequences for the Aβ_1−40_ and Aβ_1−42_ chains and varied the hydrophobic interaction energy ε_H_ from 0.4 to 0.7*k*_B_*T*. We have again simulated 100 chains in box sizes ranging from 36 × 36 × 36 to 500 × 500 × 500 lattice sites.

**Figure 7:**
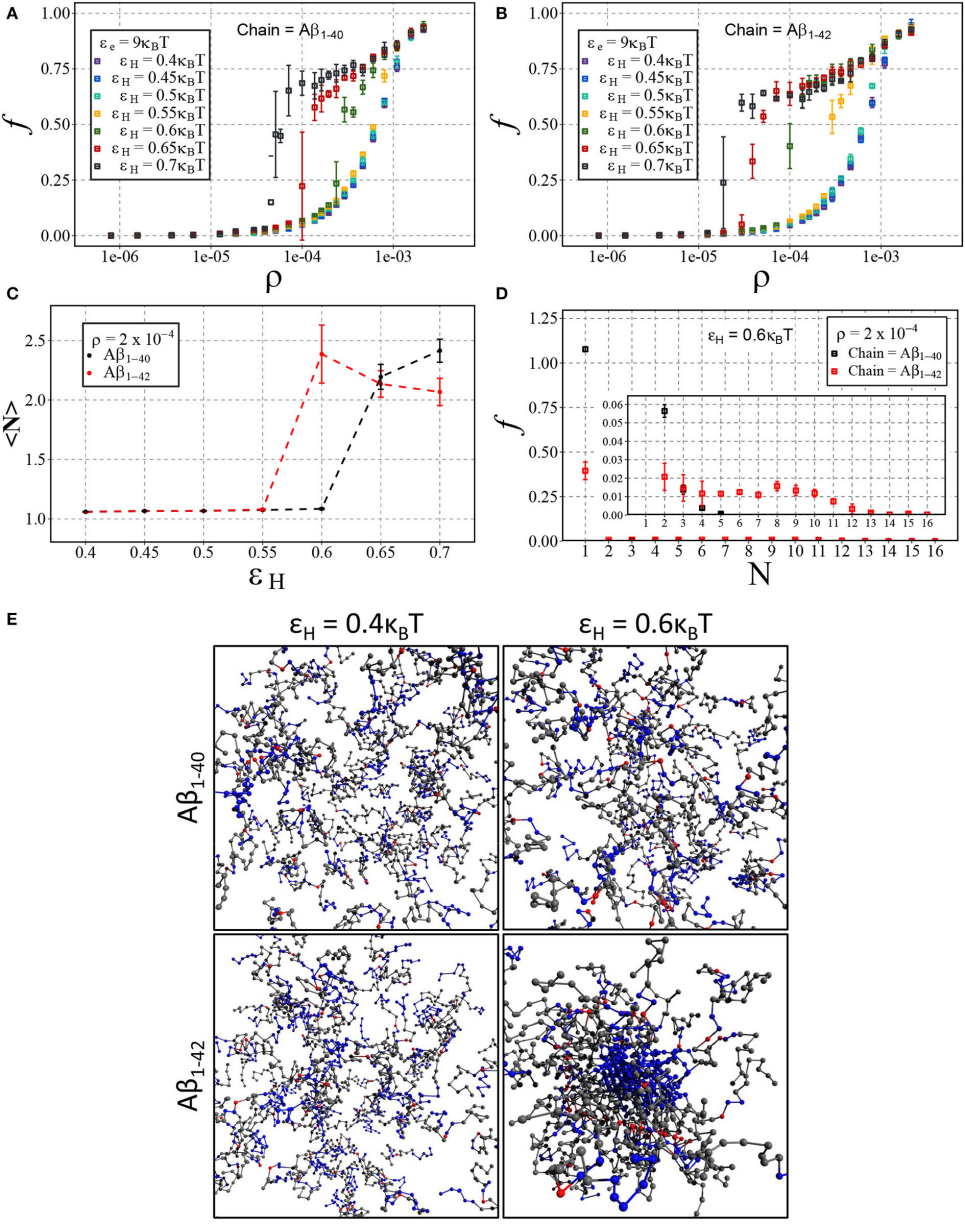
Aβ aggregation with dimerisation (nucleation) energy ε_n_ = 3*k*_B_*T* and elongation energy ε_e_ = 9*k*_B_*T*. Each point represents the mean and standard deviation of 3 simulations. The fraction of aggregated material, *f*, is plotted against the number density, ρ (in units of the lattice spacing), for varying values of ε_H_ using either **(A)** Aβ_1−40_ and **(B)** Aβ_1−42_. **(C)** Displays the change in mean cluster size, 〈*N*〉, against varying hydrophobic interaction energy, ε_H_, at a concentration of ρ = 2·10^−4^. **(D)** A size distribution plot with the fraction of material, *f*, plotted against the cluster size, *N*, with a fixed hydrophobicity of ε_H_ = 0.6*k*_B_*T* at a concentration of ρ = 2·10^−4^. **(E)** Shown are snapshots taken from the end point of the simulation for Aβ_1−40_ and Aβ_1−42_ at low (ε_H_ = 0.4*k*_B_*T*) and high (ε_H_ = 0.6*k*_B_*T*) hydrophobicity.

We have presented the results in Figures 7A,B, displaying the fraction of aggregated material *f* against the number density ρ for Aβ_1−40_ (Figure 7A) and Aβ_1−42_ (Figure 7B). In line with our results on dimerisation, we find that increasing hydrophobic interaction energy shifts the aggregation concentration to a lower concentration and that the transition to the aggregates state becomes sharp. For these higher hydrophobicities, we again find a slow ongoing increase of the fraction of aggregated material due to Ostwald ripening and/or rare events of fusion of condensates. After close inspection of Figures 7A,B, we observe that the transition occurs for Aβ_1−42_ at lower concentrations than for Aβ_1−40_, which we attribute to a larger number of hydrophobic interactions for the longer chain. Indeed, Figure 7C shows that the mean aggregate size at ρ = 2·10^−4^ sharply increases at ε_H_ = 0.6 for the longer chain and at ε_H_ = 0.65 for the shorter chain. The mean values of the aggregate size are biased by the presence of small oligomers; Figure 7D reveals the presence of aggregates with 8 or more chains.

### 3.3. Aggregation of mixed Aβ_1−40_ and Aβ_1−42_

The difference in the critical concentration for condensation of Aβ_1−40_ and Aβ_1−42_, begs the question of to what extent our simple model can capture the reported observation of Aβ_1−42_-enhanced aggregation in mixtures of Aβ_1−40_ and Aβ_1−42_ (Kumar-Singh et al., 2006; Zetterberg, 2019). To investigate this, we have carried out *in silico* mixing experiments for two different values of hydrophobicity. First, we have chosen ε_H_ = 0.4*k*_B_*T* as it previously appeared to not have led to different aggregation dynamics of Aβ_1−42_ and Aβ_1−40_. Second, we have chosen ε_H_ = 0.6*k*_B_*T*, as this value showed the largest difference in the aggregation propensity of the two chains (refer to Figure 7).

**Figure 8:**
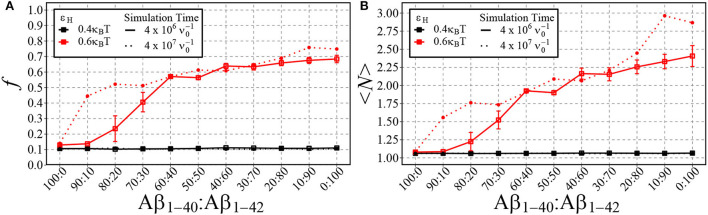
Shown are the fraction of aggregated material, *f*, **(A)** and the mean cluster size, 〈*N*〉, **(B)** using different ratios of Aβ_1−40_ and Aβ_1−42_ chains for two different values of ε_H_. 100 total chains were used in each simulation. A consistent box size of 80 × 80 × 80 (approximately 200 uM) was used in all simulations, with ε_e_ = 9*k*_B_*T* and ε_n_ = 3*k*_B_*T*. For typical simulation times (4·10^6^ in units of $\nu_{0}^{-1}$; reached in 5·10^10^ timesteps), each point represents the mean and standard error of 3 simulations. For longer simulation times (4·10^7^ $\nu_{0}^{-1}$), each data point represents the result from a single simulation.

In Figure 8, we plot the fraction of aggregated material (Figure 8A) and the mean aggregate size (Figure 8B) against the fraction of Aβ_1−42_ in a mixture of Aβ_1−42_ and Aβ_1−40_. Having in mind that for strong hydrophobicity, structural coarsening renders the system out-of-equilibrium even at long time scales, and thermal equilibrium may never be reached (refer to Figure 5), we have plotted the results after time $4\cdot 10^{6}\nu_{0}^{-1}$ and after $4\cdot 10^{7}\nu_{0}^{-1}$. As expected, for low hydrophobicity (ε_H_ = 0.4*k*_B_*T*), almost no aggregation takes place and the fraction of aggregated materials is ≈ 0.1, independently of the fraction of long chains. However, for higher hydrophobicity (ε_H_ = 0.6*k*_B_*T*) aggregation occurs rapidly for mixtures with 20% Aβ_1−42_ (solid lines), while at long timescale mixtures with 10% Aβ_1−42_ start to aggregate (dotted lines). In qualitative agreement with the experimental observations by Kuperstein et al. (2010), the critical point is strongly biased to a low fraction of Aβ_1−42_ in the mixture (< 10% in our simulations). The slow dynamics are expected, as it is typical for first-order phase separation near the critical point (Hohenberg and Halperin, 1977). However, we observe another slow process beyond the critical point, namely the increase of the aggregate size at high concentrations of Aβ_1−42_ (panel **B**). We attribute these to rare events of aggregate fusion.

## 4. Discussion and conclusion

Our study has contributed a molecular modeling approach to address (1) the observation of Aβ_1−42_-enhanced toxic plaques associated with Alzheimer's disease, despite being the less concentrated species of Aβ, and (2) the hypothesis of condensate-precursors for fibrillation. Here, we sought a molecular explanation for the different self-assembly behavior of Aβ_1−40_ and Aβ_1−42_ using sticker-and-spacer models that capture the intrinsic disorder and the effect of hydrophobicity. while the two extra amino acids in Aβ_1−42_ elongate the chain length, they also add hydrophobicity.

**Figure 9:**
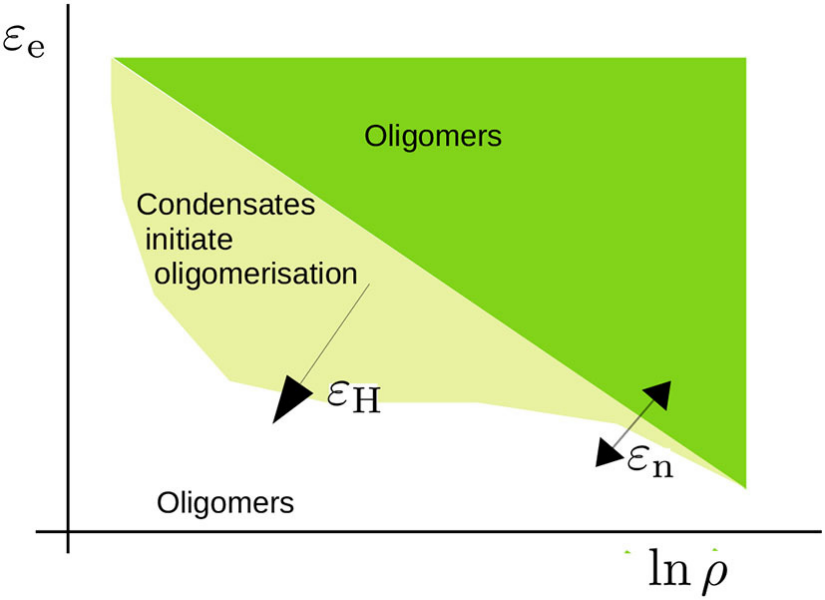
Schematic representation of the phase diagram that we have studied in the present study. The critical concentration for oligomerisation is roughly given by ln ρ_crit_ ∝ ε_e_/*k*_B_*T* with ε_e_ the elongation energy. This critical concentration is affected by excluded volume effects near the overlap concentration and leads to an apparent dependence on the nucleation energy ε_n_ (refer to Section 3.1). For a sufficiently high hydrophobic interaction energy ε_H_, condensates form. The concentration is high within these condensates and promotes oligomer formation. The shape of this region is a qualitative estimate that acknowledges that phase separation takes place within a limited concentration range.

The model predicts a rich range of phenomena due to the interplay between the nucleation and elongation of aggregates with the LLPS of condensates, driven by hydrophobic or other non-specific interactions, which we have summarised in Figure 9. The high concentration inside the condensates enhances the rate of nucleation through the formation of dimers. Furthermore, at these high concentrations the excluded-volume interactions, which in dilute conditions would hamper the growth of aggregates, are screened, such that the cooperativity of oligomerisation is enhanced. Consequently, we find a sharp transition from the unbound to the aggregated state. However, the dynamics by which aggregates may form are slow close to the critical conditions for LLPS. At concentrations above the critical concentration, the growth of aggregates is slow due to the relatively slow dynamics of Ostwald ripening and/or fusion events.

A similar secondary-nucleation mechanism was discussed previously by Cohen et al. (2013) and Michaels et al. (2020), and it was found that the rate constants for primary nucleation, elongation, and secondary nucleation are 100-, 10-, and 3-fold greater, respectively, for Aβ_1−42_ compared to Aβ_1−40_ (Meisl et al., 2014). This provides evidence that the addition of two c-terminal hydrophobic amino acids promotes aggregation propensity, in particular, the rate of primary nucleation. This is in agreement with Roche et al. (2016) who propose that the primary nucleation of Aβ is driven by non-specific hydrophobic interactions which explain the difference in the aggregation rates of Aβ_1−40_ and Aβ_1−42_.

Despite its simplicity, the model also predicts that a small fraction (< 10%) of Aβ_1−42_ in a mixture of Aβ_1−42_ and Aβ_1−40_ shifts the critical concentration for the LLPS of condensates to lower values, which in turn leads to the nucleated self-assembly of fibrillar oligomers at reduced concentrations. This finding is qualitatively consistent with Kuperstein et al. (2010) who found that even a small change from a 1:9 to a 3:7 ratio of Aβ_1−42_:Aβ_1−40_ caused a dramatic change in the aggregation kinetics and toxicity of the two mixtures *in vitro* and *in vivo*. This 3:7 ratio is of particular importance as it reflects the ratio of Aβ_1−42_:Aβ_1−40_ observed in familial AD patients (Scheuner et al., 1996) suggesting that maintaining a physiological ratio of Aβ_1−42_:Aβ_1−40_ is of great importance and may be an effective therapeutic target.

Whether Aβ_1−40_ and Aβ_1−42_ can co-fibrilise is still under debate, Cukalevski et al. (2015) found that mixing Aβ_1−40_ and Aβ_1−42_ leads to the generation of separate homomolecular fibrils. In contrast, studies have shown that mixing Aβ_1−40_ and Aβ_1−42_ leads to the formation of mixed oligomers and fibrils (Cerofolini et al., 2020; Gu and Guo, 2021). However, it is accepted that Aβ_1−40_ and Aβ_1−42_ interact at the molecular level with increased levels of Aβ_1−40_ inhibiting fibril formation and increased levels of Aβ_1−42_ promoting aggregation (Hasegawa et al., 1999; Yan and Wang, 2007; Jan et al., 2008; Pauwels et al., 2012). Despite studies disagreeing on co-fibrilisation, they agree that prefibrillar intermediates consisting of both Aβ_1−40_ and Aβ_1−42_ exist. Our findings suggest that Aβ_1−42_ interacts with Aβ_1−40_, facilitating its aggregation. When only Aβ_1−40_ chains are present and ε_H_ = 0.6*k*_B_*T*, approximately 10% of the chains are aggregated. However, with 4·10^6^ $\nu_{0}^{-1}$, when Aβ_1−40_ and Aβ_1−42_ are mixed at a 60:40 ratio, *f* increases to approximately 60%. If only Aβ_1−42_ chains are capable of aggregation, *f* = 40% at maximum. As this is not the case, it demonstrates that the addition of Aβ_1−42_ is sufficient to promote the aggregation of Aβ_1−40_ under these conditions. One explanation may be that when the more aggregation prone Aβ_1−42_ is present, it forms the primary nuclei overcoming the initial energy barrier that then enables Aβ_1−40_ to aggregate. This theory is supported by Cukalevski et al. (2015) who showed that upon mixing Aβ_1−42_ and Aβ_1−40_ it is Aβ_1−42_ that aggregates first, followed by Aβ_1−40_.

In conclusion, we have presented a simple sticker-and-spacer lattice model that captures a wide range of molecular phenomena of relevance to the literature on Aβ aggregation, which enables us to interpret those phenomena in terms of the simple concepts of nucleation-elongation models and hydrophobicity and polymeric excluded-volume effects at the level of coarse-grained sticky-polymer models. We hope this approach will lead to further (quantitative) refinements to the understanding of typical experimental time and length scales of Aβ aggregation in few-component *in vitro* and complex multi-component *in vivo* studies.

## Data availability statement

The datasets generated/analyzed presented in this study are available on Zenodo https://doi.org/10.5281/zenodo.7053937. The simulation code is available on request.

## Author contributions

CS and JC collected the data, performed the analysis, and wrote the first draft of the manuscript. CS administered the project and developed, coded the model, and the simulation algorithm. All authors conceived and designed the study, contributed to manuscript revision, read, and approved the submitted version.

## Funding

JC acknowledges financial support from the Physics of Life UK. SQ acknowledges support from Alzheimer's Research UK (RF2019-A-001). CS acknowledges support from the Engineering and Physical Sciences Research Council [Grant No. EPSRC (EP/N031431/1)].

## Conflict of interest

The authors declare that the research was conducted in the absence of any commercial or financial relationships that could be construed as a potential conflict of interest.

## Publisher's note

All claims expressed in this article are solely those of the authors and do not necessarily represent those of their affiliated organizations, or those of the publisher, the editors and the reviewers. Any product that may be evaluated in this article, or claim that may be made by its manufacturer, is not guaranteed or endorsed by the publisher.
